# Specificity of NifEN and VnfEN for the Assembly of Nitrogenase Active Site Cofactors in Azotobacter vinelandii

**DOI:** 10.1128/mBio.01568-21

**Published:** 2021-07-20

**Authors:** Ana Pérez-González, Emilio Jimenez-Vicente, Jakob Gies-Elterlein, Alvaro Salinero-Lanzarote, Zhi-Yong Yang, Oliver Einsle, Lance C. Seefeldt, Dennis R. Dean

**Affiliations:** a Department of Biochemistry, Virginia Tech, Blacksburg, Virginia, USA; b Institute of Biochemistry, Albert-Ludwigs Universität, Freiburg, Germany; c Centre for Plant Biotechnology and Genomics, Instituto Nacional de Investigación y Tecnología Agraria y Alimentaria (INIA), Universidad Politécnica de Madrid (UPM), Madrid, Spain; d Department of Chemistry and Biochemistry, Utah State University, Logan, Utah, USA; University of Arizona

**Keywords:** assembly, FeFe-cofactor, FeMo-cofactor, FeV-cofactor, molybdenum, nitrogenase, vanadium

## Abstract

The nitrogen-fixing microbe Azotobacter vinelandii has the ability to produce three genetically distinct, but mechanistically similar, components that catalyze nitrogen fixation. For two of these components, the Mo-dependent and V-dependent components, their corresponding metal-containing active site cofactors, designated FeMo-cofactor and FeV-cofactor, respectively, are preformed on separate molecular scaffolds designated NifEN and VnfEN, respectively. From prior studies, and the present work, it is now established that neither of these scaffolds can replace the other with respect to their *in vivo* cofactor assembly functions. Namely, a strain inactivated for NifEN cannot produce active Mo-dependent nitrogenase nor can a strain inactivated for VnfEN produce an active V-dependent nitrogenase. It is therefore proposed that metal specificities for FeMo-cofactor and FeV-cofactor formation are supplied by their respective assembly scaffolds. In the case of the third, Fe-only component, its associated active site cofactor, designated FeFe-cofactor, requires neither the NifEN nor VnfEN assembly scaffold for its formation. Furthermore, there are no other genes present in A. vinelandii that encode proteins having primary structure similarity to either NifEN or VnfEN. It is therefore concluded that FeFe-cofactor assembly is completed within its cognate catalytic protein partner without the aid of an intermediate assembly site.

## INTRODUCTION

Development of a eukaryotic organism having the capacity to reduce atmospheric dinitrogen (N_2_) to ammonia remains an important frontier in biological research, having the promise for profound agronomic, economic, and ecological benefit (1–3). One strategy to achieve this goal involves introduction of the genetic determinants for biological nitrogen fixation from bacteria or archaea into a eukaryotic organism. Two types of nitrogen-fixing systems have been reported in the literature: one involving oxygen-sensitive, complex, two-component metalloenzymes, referred to as the nitrogenases (4, 5), and the other involving an oxygen-insensitive superoxide-dependent enzyme (6). Although the proposed oxygen-insensitive system attracted considerable interest when first reported, more recent studies have shown that it does not exist (7). Thus, efforts to engineer eukaryotic organisms having the capacity for nitrogen fixation have focused in recent years on the canonical nitrogenase components.

**FIG 1:**
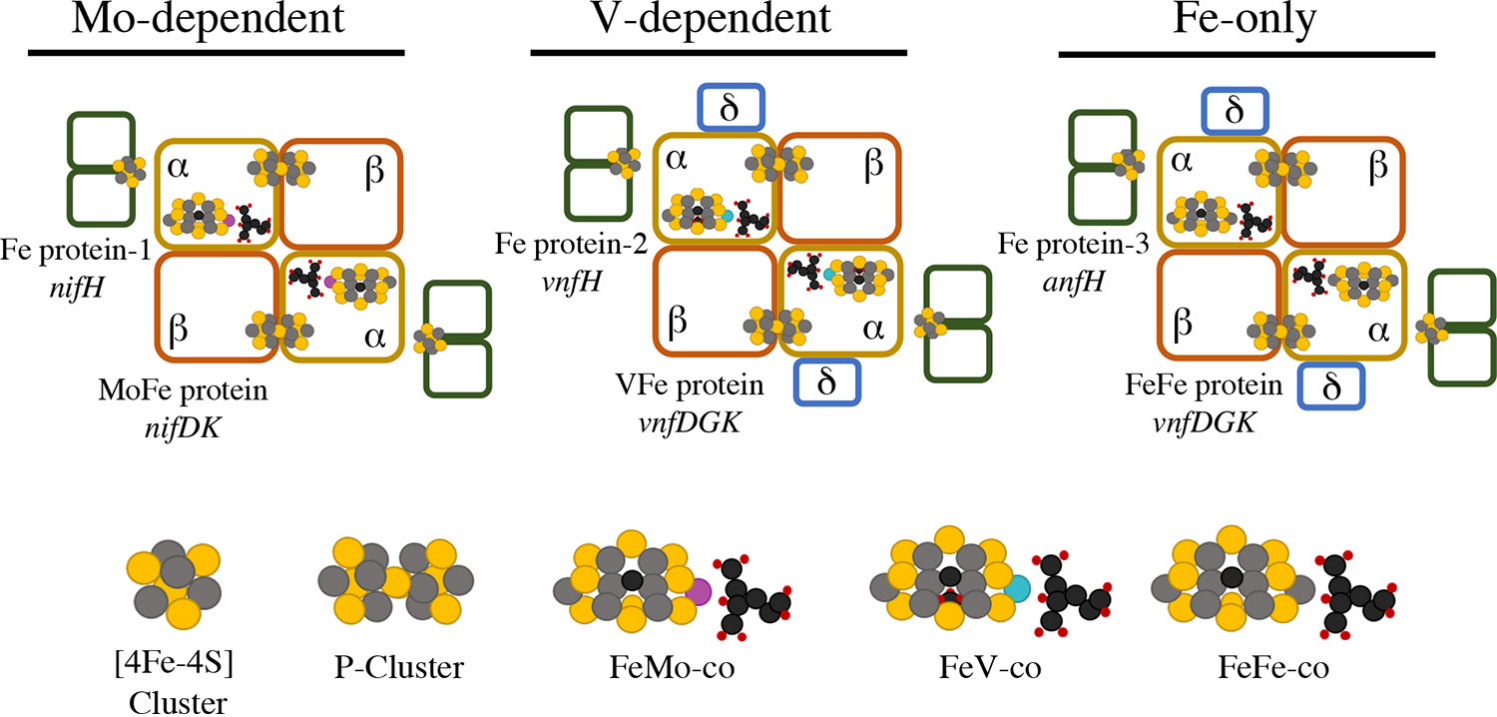
Schematic representation of the architecture of the catalytic components of the three nitrogenases expressed in A. vinelandii. Protein designations and their encoding genes are indicated below each component. Nucleotide- and reductant-dependent electron delivery from the Fe protein to the MoFe protein is accomplished by component protein interaction and nucleotide hydrolysis. The tetrameric MoFe protein contains two pairs of metalloclusters: P-cluster located at the interface of the α-β subunits and FeMo-cofactor contained entirely within the α subunits. The architecture of the VFe and the FeFe nitrogenases is similar to that of the MoFe, except for the presence of a third subunit, δ, encoded by *vnfG* and *anfG* genes, respectively. The corresponding Fe proteins for the three systems are indicated as 1, 2, or 3. Structures of FeMo-cofactor, FeV-cofactor, and FeFe-cofactor are shown in more detail in Fig. 2. Atoms in the structures are indicated as follows: yellow, sulfur; gray, iron; black, carbon; red, oxygen. The distinctive metals contained in FeMo-cofactor, FeV-cofactor, and FeFe-cofactor are represented in magenta, blue, and gray, respectively.

**FIG 2:**
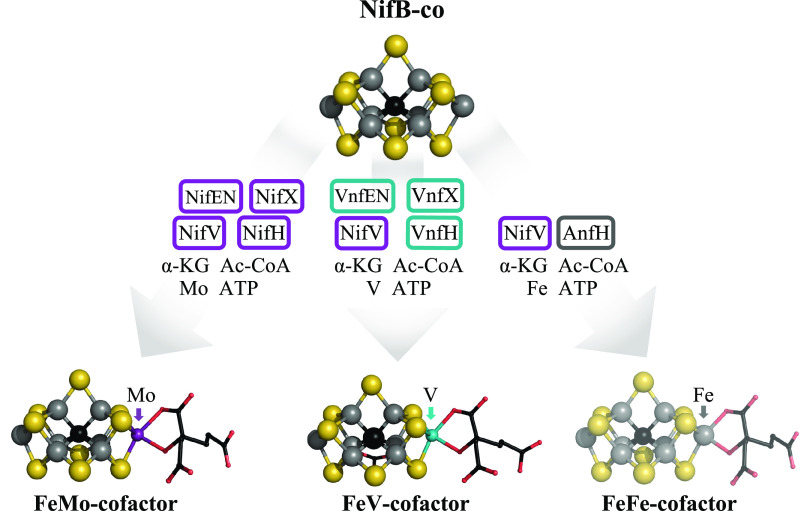
Scheme for cofactor maturation for the three nitrogenases. NifB-co, the product of NifB catalysis, is the precursor for the catalytic cofactor contained in each of the respective nitrogenase catalytic components. NifV catalyzes the condensation of acetyl coenzyme A (Ac-CoA) and α-ketoglutarate (α-KG) to form homocitrate, the organic constituent contained in each of the mature cofactors. Fe protein species associated with each system are known or inferred to be required for cofactor maturation. Maturation of FeMo-cofactor associated with the Mo-dependent nitrogenase occurs on the NifEN assembly scaffold, whereas maturation of FeV-cofactor occurs on the VnfEN assembly scaffold. NifX and VnfX are respectively considered to have nonessential roles in trafficking NifB-co to the proper assembly scaffold. Neither NifEN nor VnfEN is required for assembly of the FeFe-cofactor. Atoms in the structures are indicated as follows: black, carbon; yellow, sulfur; gray, iron; red, oxygen; magenta, molybdenum; blue, vanadium. Although not obvious in the figure, one of the bridging sulfurs present in the core of the MoFe protein is replaced by a proposed carbonate group in the FeV-cofactor (50). The coordinates for FeMo-cofactor and FeV-cofactor are from PDB files 3U7Q and 5N6Y, respectively. A structure for FeFe-cofactor has not been established by crystallographic methods but is inferred from spectroscopic and elemental analysis to be similar to FeMo-cofactor with Fe replacing Mo. This inference is represented in the figure with a lighter color for this cofactor. The NifB-co structure is derived from FeMo-cofactor with Fe replacing Mo and lacking homocitrate. Structures were visualized with PyMOL 2.4.0-Incentive product (Schrodinger, LLC).

Remarkable studies pioneered in the laboratory of Paul Bishop (8, 9) established that three genetically distinct, but structurally similar and, as recently reported, also mechanistically equivalent (10), nitrogenase isoenzymes are produced by the nitrogen-fixing proteobacterium Azotobacter vinelandii (Fig. 1 and 2). These isozymes are generically referred to as the Mo-dependent, V-dependent, and Fe-only nitrogenases, designations that reflect the identity of the metal coordinated to the organic constituent (*R*-homocitrate) within their respective active site cofactors, as well as the physiological conditions under which they are produced. Genes encoding the corresponding components have been given the trivial designations *nif* (Mo dependent), *vnf* (V dependent), and *anf* (Fe only). In the case of the Mo-dependent system, *nifH* encodes a dimeric, nucleotide- and reductant-dependent reductase called the Fe protein (also called component II). It contains a redox-active Fe_4_S_4_ cluster that supplies electrons to the tetrameric complex of the *nifD* and *nifK* gene products designated the MoFe protein (also called component I), which provides the site for N_2_ binding and reduction. The overall process involves sequential component protein association/dissociation, intercomponent electron transfers, and ATP hydrolysis. MoFe protein contains two pairs of metalloclusters: Fe_7_S_9_Mo:C:(*R*)-homocitrate designated FeMo-cofactor and an Fe_8_S_7_ P-cluster. FeMo-cofactor is the site for substrate binding and reduction whereas P-clusters mediate inter- and intracomponent delivery of electrons from the Fe protein Fe_4_S_4_ cluster to FeMo-cofactor. Structural, catalytic, and biophysical features of the nitrogenases have been recently reviewed (11–15).

**FIG 3:**
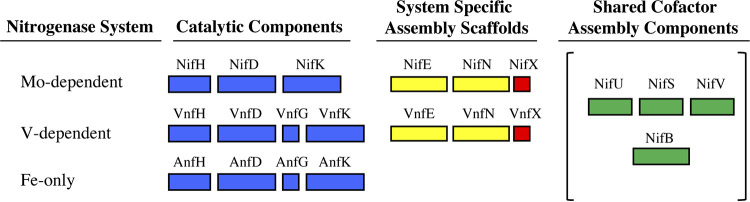
Schematic representation of the essential genes associated with the different nitrogenases expressed in A. vinelandii. The structural genes encoding nitrogenase catalytic components are filled in blue for each nitrogenase system. Four *nif* gene products (NifU, NifS, NifV, and NifB) necessary for maturation of MoFe protein, VFe protein, and FeFe protein are bracketed and indicated in green. NifEN and VnfEN gene products (in yellow) are required as the assembly nodes for cofactor formation for the MoFe and the VFe protein, respectively. The proposed system-specific carrier proteins NifX and VnfX are indicated in red.

The general architecture of the catalytic components of the V-dependent and Fe-only components is similar to the Mo-dependent components as schematically shown in Fig. 1 to 3. For example, the product of the *vnfH* gene (Fe protein-2) and product of the *anfH* gene (Fe protein-3) have functions equivalent to the product of the *nifH* gene (Fe protein-1). The products of the *vnfDGK* genes (VFe protein) and products of the *anfDGK* genes (FeFe protein) have functions equivalent to the products of the *nifDK* genes (MoFe protein). One significant difference is the presence of an additional δ subunit in the VFe protein and FeFe protein compared to the MoFe protein (16–18). Another difference involves the metal composition of the corresponding active site cofactors (FeMo-cofactor, FeV-cofactor, and FeFe-cofactor) associated with the different components (Fig. 1 and 2). Which nitrogenase system operates under a particular physiological condition depends upon the availability of Mo or V in the growth medium and reflects the relative catalytic efficiency of the three systems. Namely, the Mo-dependent system is preferred when Mo is available because it is the most efficient one and the V-dependent system is preferred when Mo is scarce but V is available, because the V-dependent system is more efficient than the Fe-only system (5, 19).

The complex nature of nitrogenase catalysis, which is not fully understood, is also reflected in the number and organization of genes associated with the process. In addition to genes encoding the catalytic components, there are numerous other genes/products involved in various aspects such as formation and insertion of the associated metalloclusters, oxygen protection, coupling electron transfer to cellular metabolism, and regulation of gene expression (15). The functions of many of the gene products associated with the various nitrogenases are not known, and many of them are not necessary to support diazotrophic growth under standard laboratory conditions. In the case of the Mo-dependent nitrogenase, including the structural components and excluding regulatory components, there are 7 gene products (Fig. 3) required for the synthesis of FeMo-cofactor prior to its insertion to form an active MoFe protein (20). These include NifU and NifS, involved in the initial formation of Fe-S cluster building blocks necessary for metallocluster assembly, and NifB, NifV, NifH, and NifEN, which have specific functions in the formation of FeMo-cofactor. Early studies demonstrated that FeMo-cofactor is separately formed and inserted into an immature form of the MoFe protein called apo-MoFe protein (21), which contains intact P-clusters but no FeMo-cofactor (22, 23). There are two assembly nodes involved in that process. One node is provided by NifB, a radical *S*-adenosylmethionine (SAM)-dependent enzyme responsible for formation of an Fe_8_S_9_:C core, designated NifB-co (15, 24). The other assembly node is provided by an α_2_β_2_ NifEN complex that has structural features similar to the MoFe protein (25–27). The NifEN complex is the terminal assembly scaffold upon which NifB-co is converted to FeMo-cofactor through the action of NifV (homocitrate synthase) and Fe protein, in a process that requires Mo and ATP (Fig. 2) (26, 28, 29). NifX, NifY, and NafY are proposed to be nonessential metallocluster trafficking proteins involved in shuttling NifB-co to NifEN (NifX function) and FeMo-cofactor to apo-MoFe protein (NafY/NifY). Although NifX, NifY, and NafY are dispensable for FeMo-cofactor formation, their function has been inferred by their ability to bind NifB-co or FeMo-cofactor and a corresponding ability to bind either apo-MoFe protein or the NifEN complex (15, 30).

NifU, NifS, NifV, and NifB have the same function for formation of FeV-cofactor as they have for FeMo-cofactor formation, thereby indicating some functional cross talk in the respective metallocluster assembly processes (9, 31). However, in the case of the FeV-cofactor formation, the function of the NifEN complex is replaced by a proposed VnfEN complex and the proposed NifB-co trafficking partner NifX appears to be replaced by VnfX. Also, the function of NifH (Fe protein-1) is replaced by VnfH (Fe protein-2) in maturation of VFe protein. It should be noted, however, that both *in vitro* and *in vivo* studies have demonstrated that VnfH can substitute for the function of NifH for the formation of FeMo-cofactor (32, 33). It has also been claimed that NifEN can replace the function of VnfEN for formation of FeV-cofactor (34, 35). These findings present a conundrum concerning how the respective systems can provide specificity for insertion of the correct metal, Mo or V, into their corresponding FeMo-cofactor or FeV-cofactor, leading to one aspect of the current work. Namely, can NifEN indeed replace the function of VnfEN in the assembly of FeV-cofactor?

It is also known that NifU, NifS, NifV, and NifB have the same function for formation of FeFe-cofactor as they have for FeMo-cofactor and FeV-cofactor (9, 31). Gene/products that have a common function in the formation of all three cofactors are bracketed in Fig. 3. It is presumed, although not experimentally established, that AnfH (Fe protein-3) has the same function in FeFe-cofactor formation that NifH and VnfH have in FeMo-cofactor and FeV-cofactor formation, respectively. Based on mutagenesis studies, and the observation that there are no apparent Fe-only system analogs to NifEN and VnfEN (Fig. 3) it has been suggested that either NifEN or VnfEN must function as a terminal assembly node for the processing of NifB-co to form FeFe-cofactor (35). In contrast, Yi-Ping Wang, Ray Dixon, and colleagues have reported that an active FeFe nitrogenase can be produced in Escherichia coli by heterologous expression of the structural components together with expression of only *nifU*, *nifS*, *nifV*, and *nifB* (36). In other words, neither NifEN nor VnfEN is required to form an active FeFe nitrogenase in the E. coli-based system, indicating that conversion of NifB-co to FeFe-cofactor might occur within an immature form of the FeFe protein without involvement of an intermediate cofactor assembly complex. These differences can be reconciled only if either the heterologous expression system results in an anomalous maturation process or the proposed requirement for NifEN or VnfEN for formation of FeFe-cofactor in the native host is incorrect, possibly as a result of a physiological regulatory anomaly related to strain constructions. This is an important issue to resolve as it relates to confidence in assessing the minimal requirement for producing an active nitrogen-fixing system in a eukaryote. In this regard it should be noted that the Fe-only system is particularly attractive for this purpose because it requires Fe as the only transition metal necessary for formation of an active nitrogenase, and consequently, systems for the acquisition and activation of either V or Mo are not necessary. For these reasons we have reinvestigated the possible requirement of either NifEN or VnfEN for the *in vivo* maturation of FeFe protein.

## RESULTS

**FIG 4:**
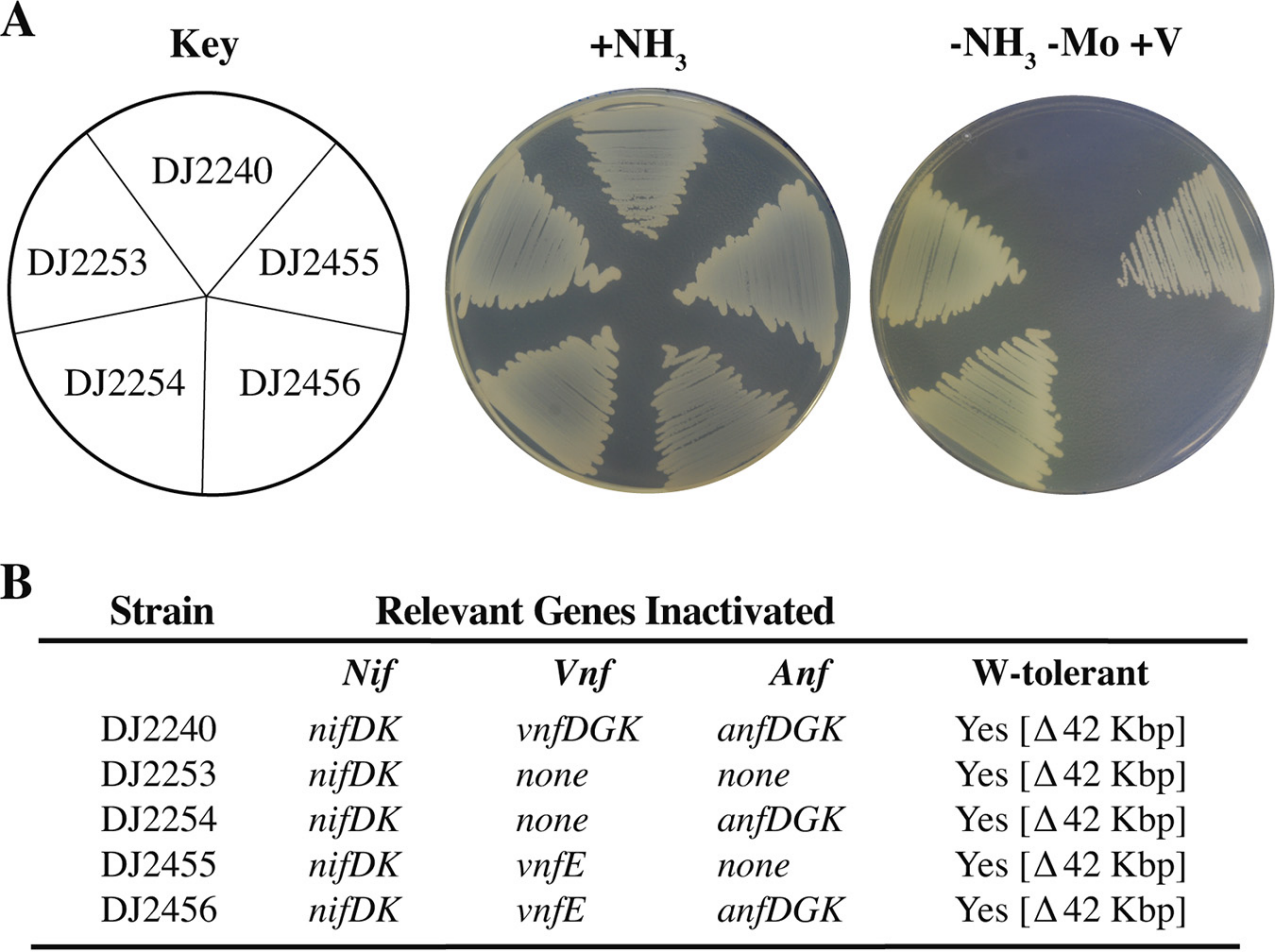
NifEN cannot substitute for the function of VnfEN. Strains expressing VFe nitrogenase were cultured on Mo-free Burks medium agar plates containing a fixed nitrogen source (+NH_3_) or under diazotrophic conditions in a medium supplemented with vanadium but not Mo (-NH_3_ -Mo +V) (A). Relevant genotypes are listed in panel B and Table S1. W-tolerance indicates the strain carries a 42,096-bp deletion in the A. vinelandii genome that includes genes whose products are involved in Mo acquisition (37, 55). The key experimental observation involves comparison of the growth of strains DJ2455 and DJ2456.

As a first step to test if NifEN can physiologically replace the function of VnfEN for formation of FeV-cofactor, a W-tolerant strain (DJ2253) unable to effectively transport Mo (37) and also deleted for genes encoding the MoFe protein subunits was isolated (see Tables S1 and S2 in the supplemental material). Using a strain deficient in Mo acquisition as a result of W tolerance to prevent the very effective repression of V-dependent and Fe-only nitrogenases by trace levels of Mo in the culture medium is the same approach as used by Eady and colleagues for efficient expression and purification of the V-dependent nitrogenase from Azotobacter chroococcum (38). A. vinelandii strain DJ2253 can grow in the absence of fixed nitrogen in medium supplemented with V (Fig. 4) and therefore has an intact V-dependent nitrogenase. Purification and biophysical characterization of VFe protein produced by DJ2253 have been previously described in detail (39). A derivative of DJ2253 inactivated for VnfEN (DJ2455) retains the capacity for diazotrophic growth (Fig. 4). The genotype of this strain, with the exception of the inclusion of W-tolerance, and the corresponding phenotype are generally equivalent to previously published work and, therefore, nominally in agreement with the proposal that NifEN can replace the function of VnfEN. Nevertheless, the possibility remained that the retention of diazotrophic growth by DJ2455 having VnfEN inactivated could be the result of expression of the Fe-only system in this particular construct rather than accumulation of active VFe nitrogenase. This possibility was confirmed by showing that a capacity for diazotrophic growth is lost by a strain (DJ2456) for which both VnfEN and Fe-only nitrogenase are inactivated (Fig. 4). Thus, diazotrophic growth by a strain inactivated for VnfEN observed in the present work is actually the result of Fe-only nitrogenase activity rather than NifEN-directed FeV-cofactor formation. From these observations it is concluded that NifEN does not physiologically replace the function of VnfEN in the maturation of FeV-cofactor. This conclusion is in agreement with the observation of VnfEN being essential for the V nitrogenase in Anabaena variabilis (40), and it is also supported on the basis of transcriptome analyses (41). Namely, culturing A. vinelandii in the absence of Mo and in the presence of V does not stimulate expression of *nifEN* above basal levels, indicating that a level of NifEN sufficient to support FeV-cofactor formation is unlikely to accumulate in cells grown in the presence of V. Previous studies have already established that a strain inactivated for NifEN produces an inactive FeMo-cofactor-less MoFe protein, indicating that VnfEN cannot physiologically substitute for NifEN (26). There is also biochemical and genetic evidence that neither the NifEN nor VnfEN scaffolds can functionally replace each other such that the proper heterometal becomes incorporated into the appropriate cofactor. Namely, FeV-cofactor can be inserted into MoFe protein and FeMo-cofactor can be inserted into the VFe protein, but in both cases misincorporation of the proper heterometal results in a “hybrid” species with altered catalytic properties. That is, these “hybrid” enzymes cannot effectively reduce N_2_, but they are competent in reducing certain other substrates (42–46). Moreover, surveillance of microbial genomes that encode V-dependent nitrogenase catalytic components found that they also encode *vnfEN* counterparts, further indicating a requirement for VnfEN in providing heterometal specificity for formation of FeV-cofactor (47).

10.1128/mBio.01568-21.3 TABLE S1 List of A. vinelandii strains used in this work. The list includes the detailed genotypes for each strain used in this work. Information in the table permits tracing of the genealogy of each strain back to the original DJ and CA wild-type strains. Details of plasmids used for the construction of each strain can be found in Table S2. DJ2486 is a pseudorevertant originated from DJ2475. W^T^, tungsten tolerant; H-TAG, His tag; S-TAG, Strep-tag; km, kanamycin; sm, spectinomycin; gm, gentamicin. Download Table S1, DOCX file, 0.02 MB. Copyright © 2021 Pérez-González et al. 2021 Pérez-González et al. https://creativecommons.org/licenses/by/4.0/ This content is distributed under the terms of the Creative Commons Attribution 4.0 International license.

10.1128/mBio.01568-21.4 TABLE S2 List of plasmids used for A. vinelandii strain construction. Location of residues removed and/or placement of insertions is indicated. Download Table S2, DOCX file, 0.01 MB. Copyright © 2021 Pérez-González et al. 2021 Pérez-González et al. https://creativecommons.org/licenses/by/4.0/ This content is distributed under the terms of the Creative Commons Attribution 4.0 International license.

**FIG 5:**
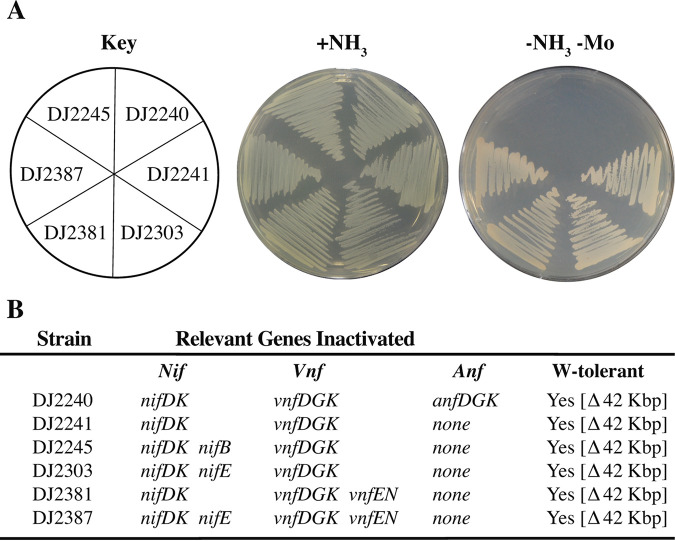
Neither NifEN nor VnfEN is required for Fe-only system-dependent diazotrophic growth. Strains expressing FeFe nitrogenase were cultured on Burks medium agar plates containing a fixed nitrogen source (+NH_3_) or under diazotrophic growth conditions without the addition of Mo (-NH_3_ -Mo) (A). Relevant genotypes are listed in panel B and Table S1. W-tolerance indicates the strain carries a 42,096-bp deletion in the A. vinelandii genome that includes genes whose products are involved in Mo acquisition (37, 55).

To assess whether or not either NifEN or VnfEN is required for formation of an active Fe-only nitrogenase, strains inactivated for both the MoFe protein and VFe protein and also inactivated for either NifEN or VnfEN, separately and in combination (DJ2303, DJ2381, UF63, UF64, and DJ2387), were independently isolated in two different laboratories and tested for their diazotrophic growth phenotypes (Fig. 5; see also Fig. 9). Positive- and negative-control experiments involved construction of a strain (DJ2240) inactivated for all three nitrogenase systems, a strain inactivated for both the MoFe protein and the VFe protein but having NifEN and VnfEN intact (DJ2241), and a strain (DJ2245) having MoFe protein, VFe protein, and NifB inactivated. The ability of each of these strains to grow on nitrogen-free medium that contains neither a Mo nor a V supplement is shown in Fig. 5 (see also Fig. 9). Control strains inactivated for all three nitrogenase catalytic components, or one having NifB inactivated, exhibit no capacity for diazotrophic growth. The requirement for NifB to support formation of an active Fe-only nitrogenase confirms the requirement for its product, NifB-co, for formation of FeFe-cofactor (Fig. 5). A strain producing an intact Fe-only system (DJ2241) or those having either NifEN inactivated (DJ2303, UF63), VnfEN inactivated (DJ2381, UF64), or both NifEN and VnfEN inactivated (DJ2387) all show comparable levels of diazotrophic growth capacity (Fig. 5; see also Fig. 9). From these data it is concluded that neither NifEN nor VnfEN provides an essential assembly node for formation of FeFe-cofactor.

**FIG 6:**
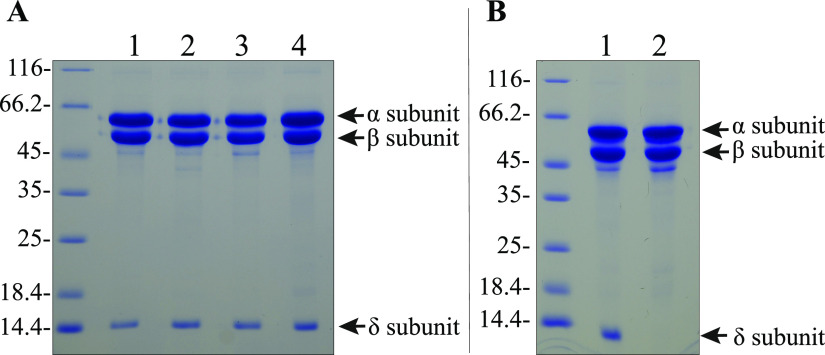
SDS-PAGE of FeFe protein purified from different strains. (A) DJ2241 (wild type, lane 1); DJ2303 (inactivated for NifE, lane 2); DJ2381 (inactivated for VnfEN, lane 3); DJ2387 (inactivated for NifE and VnfEN, lane 4). (B) DJ2241 (wild type, lane 1); DJ2245 (inactivated for NifB, lane 2). “Wild-type” refers to the strain having NifEN, VnfEN, and NifB intact. The relevant genotype for each strain is shown in Fig. 5B. Protein identities are indicated by arrows. Note that the δ subunit is not associated with FeFe protein produced in cells deficient in NifB-co formation (panel B, lane 2). Standards located to the left of panel A and panel B indicate molecular weights in kDa.

Considering an interest in transferring the Fe-only nitrogenase system to eukaryotic organisms, it was also important to establish that FeFe proteins produced in the absence of NifEN and VnfEN have comparable compositions, catalytic activities, metal contents, and biophysical properties. In other words, it was important to show that neither NifEN nor VnfEN has any direct biochemical role in the formation of a fully active Fe-only system. For these experiments the FeFe protein for each strain carried a Strep tag located at the C terminus of the α-subunit to enable rapid affinity purification (30). Catalytic activities reported here for Strep-tagged FeFe protein are in good agreement with a prior report for nontagged FeFe protein purified by conventional chromatographic methods (48). FeFe proteins produced from various genetic backgrounds were purified to homogeneity and characterized. All FeFe proteins produced in the presence or absence of NifEN and/or VnfEN have the same α_2_β_2_δ_2_ compositions based on SDS-PAGE analysis (Fig. 6); approximately the same catalytic activity profiles with respect to acetylene, proton, and N_2_ reduction (Table 1); and similar metal compositions (Table 2), with only minor amounts of Mo and V present. For active FeFe proteins characterized in the present work, there is an average Fe content of 27 Fe for each FeFe protein hexamer, which compares favorably with the theoretical 32 Fe for each FeFe protein assuming a full complement of both P-clusters and FeFe-cofactors. In contrast, FeFe protein produced by a NifB-deficient strain has no catalytic activity and only 15 Fe for each FeFe protein hexamer, consistent with it having only P-clusters and no catalytic FeFe-cofactor. Furthermore, as shown in Fig. 6B, FeFe protein isolated from a NifB-deficient strain does not contain the δ subunit, a feature also shared by the VFe protein produced by a NifB-deficient strain (39).

**TABLE 1:** Specific activities of FeFe proteins isolated from different genetic backgrounds

FeFe protein	Product	nmol of C_2_H_4_/min/mg	nmol of H_2_/min/mg			nmol of NH_3_/min/mg
	Substrate	0.1 atm C_2_H_2_	0.1 atm C_2_H_2_	1 atm Ar	1 atm N_2_	1 atm N_2_
DJ2241		167 ± 3	666 ± 19	878 ± 46	452 ± 29	167 ± 3
DJ2303 (*ΔnifE*)		162 ± 2	634 ± 8	849 ± 50	423 ± 8	162 ± 4
DJ2381 (*ΔvnfEN*)		162 ± 4	697 ± 11	1,018 ± 18	561 ± 4	177 ± 3
DJ2387 (*ΔnifE ΔvnfEN*)		160 ± 2	683 ± 11	1,005 ± 22	557 ± 4	173 ± 2

**TABLE 2:** Determination of iron, molybdenum, and vanadium content in FeFe proteins coming from the different genetic backgrounds^*a*^

FeFe protein	Metal content (mol/mol protein)		
	Iron	Molybdenum	Vanadium
DJ2241	26 ± 1.7	0.02 ± 0.01	0.06 ± 0.03
DJ2303 (*ΔnifE*)	26 ± 1.6	0.01 ± 0.01	0.09 ± 0.08
DJ2381 (*ΔvnfEN*)	28 ± 0.9	0.01 ± 0.01	0.09 ± 0.07
DJ2387 (*ΔnifE ΔvnfEN*)	29 ± 2.5	0.004 ± 0.002	0.10 ± 0.08
DJ2245 (*ΔnifB)*	15 ± 0.6	0.001 ± 0.001	0.03 ± 0.05

a Metal contents were quantified by ICP-MS. Molar ratios were calculated based on the molecular weight of the FeFe protein α_2_β_2_δ_2_ complex, or α_2_β_2_ complex in the case of the strain inactivated for NifB. Data presented are the average from at least two independent determinations.

**FIG 7:**
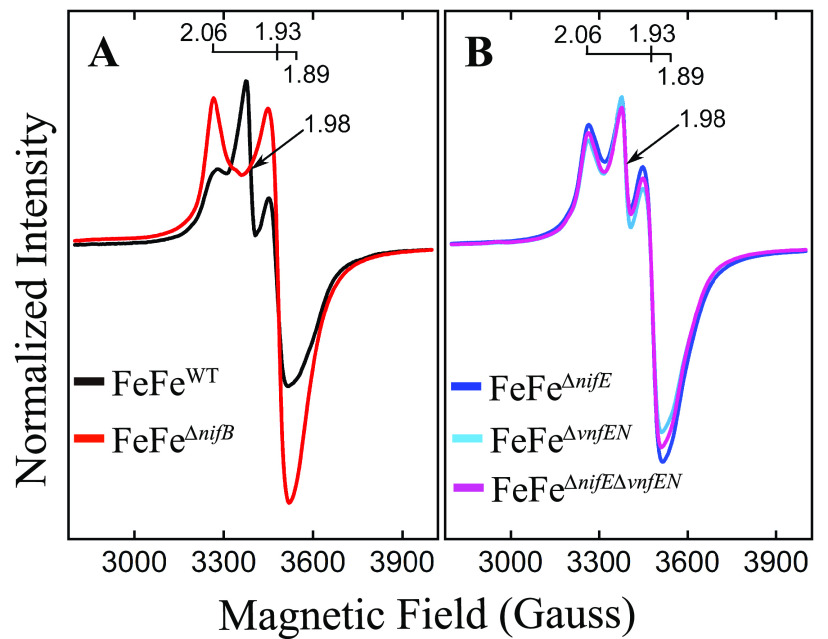
X-band EPR spectra of resting-state FeFe proteins purified from the different genetic backgrounds. All samples are Na_2_S_2_O_4_ reduced. Both panels show the S = 1/2 P-cluster associated species (g = 2.06, g = 1.93, and g = 1.89). The g = 1.98 species associated with a putative FeFe-cofactor precursor, perhaps oxidized NifB-co, is indicated by an arrow. (A) Comparison of EPR spectra of FeFe protein produced by wild type (black trace) and FeFe protein produced by a NifB-deficient strain (red trace). Notice that FeFe protein produced by a NifB-deficient strain lacks the g = 1.98 signal associated with a proposed FeFe cofactor precursor. (B) Comparison of EPR spectra of FeFe proteins isolated from strains inactivated for NifEN, VnfEN, or both NifEN and VnfEN. EPR conditions are described in Materials and Methods. Inspection of the low-field region of EPR spectrum of wild-type FeFe protein shown in Fig. S1 also reveals inflections that are not present in FeFe protein produced in a NifB-deficient background, indicating that they could be species associated with FeFe cofactor or one of its precursors. However, these low-field species represent minor components of the EPR spectrum and are variable in intensity for different wild-type FeFe protein preparations.

Electron paramagnetic resonance (EPR) spectroscopic analysis has been frequently used for the characterization of paramagnetic metal centers contained in nitrogenase components (14). Previous EPR studies revealed that both intact FeFe-cofactor and FeFe protein-associated P-cluster are EPR silent and diamagnetic (S = 0) in the dithionite-reduced, resting state (49). The black trace in Fig. 7A shows the high-field spectrum of the FeFe protein produced by strain DJ2241. There are two notable features of this spectrum. The first is a rhombic S = 1/2 feature having g values of 2.06, 1.93, and 1.89. A very similar EPR signal has also been recognized at low population in VFe protein (39, 50, 51), although its relevance to catalysis remains to be established. The other feature is a near-axial g = 1.98 species which is not present in FeFe protein prepared from a strain deficient in NifB (Fig. 7A, red trace, and Fig. S1). The full feature of this g = 1.98 species was not revealed due to overlap the rhombic S = 1/2 feature (g = 2.06, 1.93, and 1.89). However, a species having a similar line-shape and g value has also been reported for an oxidized form of NifB-co bound to NifEN (52). Based on these observations, it is speculated that the g = 1.98 species present in isolated FeFe protein is likely to represent either oxidized NifB-co or some other FeFe-cofactor precursor. The species might not originate from intact FeFe-cofactor because preliminary experiments have indicated it is not redox active under turnover conditions. The possible presence of immature P-clusters or FeFe-cofactor contained in FeFe protein samples characterized in the present work is not necessarily surprising given that the affinity purification procedure used for FeFe protein isolation would not discriminate between mature and incompletely processed forms, both of which must be present at some level in actively growing nitrogen-fixing cells. Although the EPR active species in isolated FeFe protein might not be directly involved in catalysis, they are present in approximately equal amounts in all samples regardless of whether or not either or both NifEN and VnfEN have been inactivated (Fig. 7B). This result indicates that there is no spectroscopic evidence to suggest that FeFe protein isolated from strains inactivated for either or both NifEN and VnfEN is any different than FeFe protein isolated from the parental wild-type strain.

10.1128/mBio.01568-21.1 FIG S1 Expanded EPR spectrum from Fig. 7A that shows both low- and high-field regions. Note the minor inflections in the low-field region present in wild-type FeFe protein that are not evident in the FeFe protein produced by a strain that is inactivated for NifB. The low-field signals present in the preparation of wild-type FeFe protein shown here vary in intensity, often being barely detectable in different preparations. Download FIG S1, EPS file, 1 MB. Copyright © 2021 Pérez-González et al. 2021 Pérez-González et al. https://creativecommons.org/licenses/by/4.0/ This content is distributed under the terms of the Creative Commons Attribution 4.0 International license.

**FIG 8:**
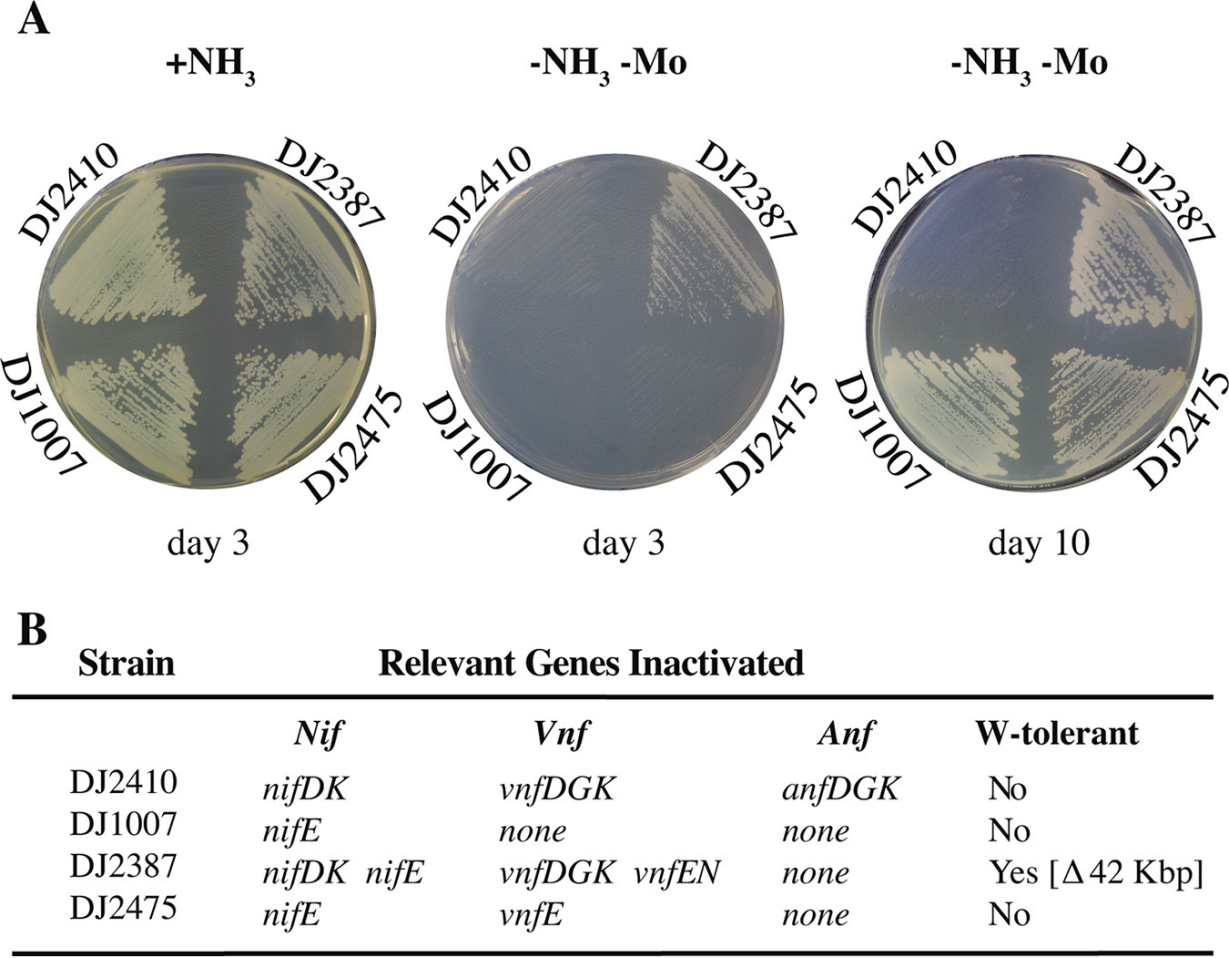
Fe-only nitrogenase-dependent diazotrophic growth for a strain disabled for FeMo-cofactor and FeV-cofactor formation shows a delayed diazotrophic growth phenotype when the Mo-acquisition system is not impaired. Strains were cultured on Mo-free Burks medium agar plates containing a fixed nitrogen source (+NH_3_) or under diazotrophic conditions without the addition of Mo (-NH_3_ -Mo) and incubated at 30°C for 3 or 10 days, as indicated (A). The key observation is that when Mo acquisition is intact, strains having FeMo-cofactor or FeV-cofactor formation disabled show a delayed growth phenotype. In contrast, strain DJ2387, for which FeMo-cofactor and FeV-cofactor formation was disabled (and which is also defective in Mo acquisition), exhibits diazotrophic growth after only 3 days. Relevant genotypes are listed in panel B and Table S1. W-tolerance indicates the strain carries a 42,096-bp deletion in the A. vinelandii genome that includes genes whose products are involved in Mo acquisition (37, 55).

**FIG 9:**
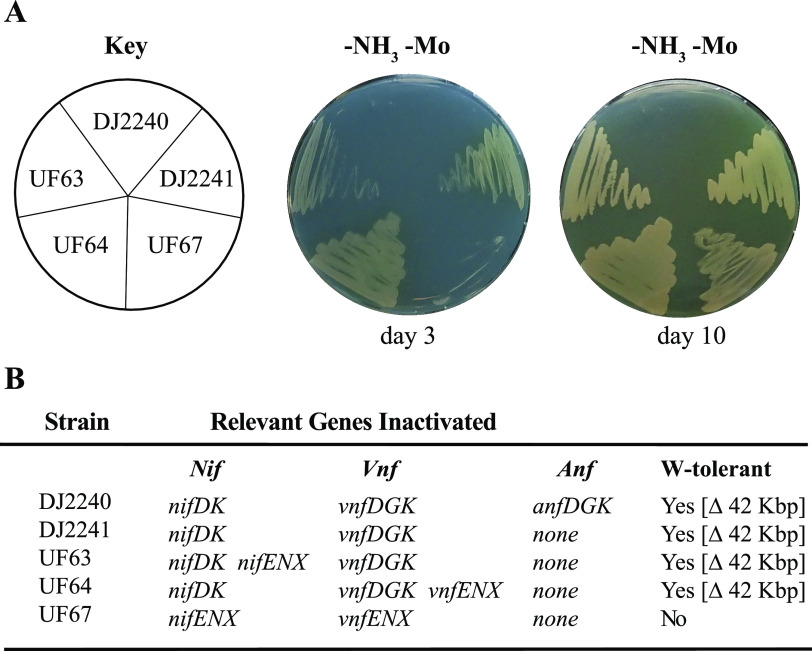
Fe-only nitrogenase-dependent diazotrophic growth for a strain having both *nifENX* and *vnfENX* genes deleted shows a delayed diazotrophic growth phenotype when the Mo-acquisition system is not impaired. Strains expressing FeFe nitrogenase were cultured on Mo-free Burks medium agar plates containing a fixed nitrogen source (+NH_3_) or under diazotrophic conditions without the addition of Mo (-NH_3_ -Mo) (A). When the Mo-acquisition system is intact, a strain having a combination of the *nifENX* and *vnfENX* genes inactivated (UF67) shows a delayed growth phenotype. Results similar to the ones obtained for the tungsten-tolerant strains DJ2303 and DJ2381 (Fig. 5) are also observed in this figure (UF63 and UF64). Relevant genotypes are listed in panel B and Table S1. W-tolerance indicates the strain carries a 42,096-bp deletion in the A. vinelandii genome that includes genes whose products are involved in Mo acquisition (37, 55).

As already noted, Fe-only nitrogenase-dependent diazotrophic growth phenotypes reported here by strains inactivated for either or both NifEN and VnfEN are different from a previous report which concluded that a functional NifEN or VnfEN is required for FeFe protein maturation (34). The expression of both V-dependent and Fe-only nitrogenases is extremely sensitive to the presence of nanomolar concentrations of Mo (53, 54). For this reason, W-tolerant strains defective in Mo acquisition or Mo-mediated repression are often used to obviate the need to scrub trace levels of Mo from the culture medium (39) when producing V-dependent or Fe-only nitrogenase systems for biochemical analyses. The primary difference in the corresponding strain constructions is that the former work involved using strains that were not defective in Mo acquisition whereas strains used in the present work are defective in Mo acquisition as a consequence of a 42,096-bp genomic deletion that includes, among others, *modE1* (55). Loss of *modE1* function is known to affect both high-affinity Mo acquisition (56, 57) and Mo-dependent repression of Fe-only nitrogenase expression (58). We therefore explored the possibility that strains inactivated for either or both NifEN and VnfEN, but otherwise having an intact Mo acquisition capability, have an impaired Fe-only-dependent diazotrophic growth capacity owing to an inability to produce sufficient FeFe protein to support effective diazotrophic growth rather than an inability to form FeFe-cofactor. In other words, could the results of the previous report be reproduced and reconciled with the results reported here? Strains inactivated for either or both NifEN and VnfEN but having no impairment in Mo acquisition were therefore independently constructed in two different laboratories (Fig. 8 and 9), and their diazotrophic growth capacities were examined. Strains inactivated for NifEN (DJ1007) or inactivated for both NifEN and VnfEN (DJ2475, UF67), but having an intact Mo regulon, exhibit no growth after 3 days of incubation, a result that is in full agreement with the original report for similar genetic constructs (34). However, after 10 days these same strains show an evident capacity for delayed diazotrophic growth (Fig. 8 and 9). By comparison, a control strain (DJ2387) inactivated for both NifEN and VnfEN, but also deficient in Mo acquisition, shows readily apparent Fe-only nitrogenase-dependent diazotrophic growth already after only 3 days (Fig. 8). It is noted that diazotrophic growth of DJ2387 can be attributed only to Fe-only nitrogenase because both the Mo-dependent and V-dependent structural components are inactivated in DJ2387. Thus, a sufficient amount of FeFe protein necessary to sustain diazotrophic growth can be produced in the absence of both NifEN and VnfEN whether or not Mo acquisition has been disabled, confirming that neither scaffold is required for maturation of the FeFe protein.

In the case of delayed Fe-only nitrogenase-dependent diazotrophic growth by strains inactivated for both NifEN and VnfEN but having an intact capacity for Mo acquisition, it was of interest to explore if this phenotype is the result of a low level of active FeFe protein or a low level of maturation. To distinguish between these possibilities, large-scale affinity purification of FeFe protein produced by a strain inactivated for both NifEN and VnfEN and having an intact capacity for Mo acquisition (DJ2479 [see Fig. S2 and Table S1 in the supplemental material]) was attempted. Almost no FeFe protein subunits could be purified in this way. In contrast, large-scale affinity purification of inactive cofactor-less FeFe protein produced in a strain inactivated for NifB, but also disabled for Mo acquisition, resulted in excellent yields (Fig. 6B, lane 2). Thus, the delayed Fe-only nitrogenase diazotrophic growth phenotype for strains DJ2475, UF67, and DJ2479 is the result of low accumulation of FeFe protein rather than a defect in maturation associated with FeFe-cofactor formation. In other words, an inability to form FeFe-cofactor does not result in the low accumulation of FeFe protein subunits.

10.1128/mBio.01568-21.2 FIG S2 (A) Strains expressing the Fe-only system that are inactivated for both the Mo-dependent and V-dependent systems but have an intact Mo-acquisition capacity exhibit a delay in diazotrophic growth. (B) The delayed diazotrophic growth phenotype exhibited by DJ2479 (inactivated for NifEN and VnfEN) is rescued by incorporation of an in-frame deletion in *modE1* (DJ2491) and also results in a W-tolerant diazotrophic growth capacity. Relevant genotypes are listed in panel C and Table S1. Download FIG S2, TIF file, 1.6 MB. Copyright © 2021 Pérez-González et al. 2021 Pérez-González et al. https://creativecommons.org/licenses/by/4.0/ This content is distributed under the terms of the Creative Commons Attribution 4.0 International license.

The original CA6 W-tolerant strain isolated in the laboratory of Bishop (37) and transferred to certain strains used in the present work (Table S1) carries a 42,096-bp deletion that removes many genes associated with Mo acquisition (55), including *modE1*. ModE1 is a Mo sensor/regulatory protein whose inactivation has been shown to result in a defect in high-affinity Mo acquisition (see Fig. 5A in reference 56), as well as partial relief of Mo-dependent repression of Fe-only nitrogenase expression (58). Based on these considerations, we conclude that the delayed Fe-only nitrogenase-dependent growth phenotype for strains inactivated for both NifEN and VnfEN, and also having an intact capacity for Mo acquisition, is likely to be associated with the repression of Fe-only nitrogenase expression associated with trace levels of Mo.

**FIG 10:**
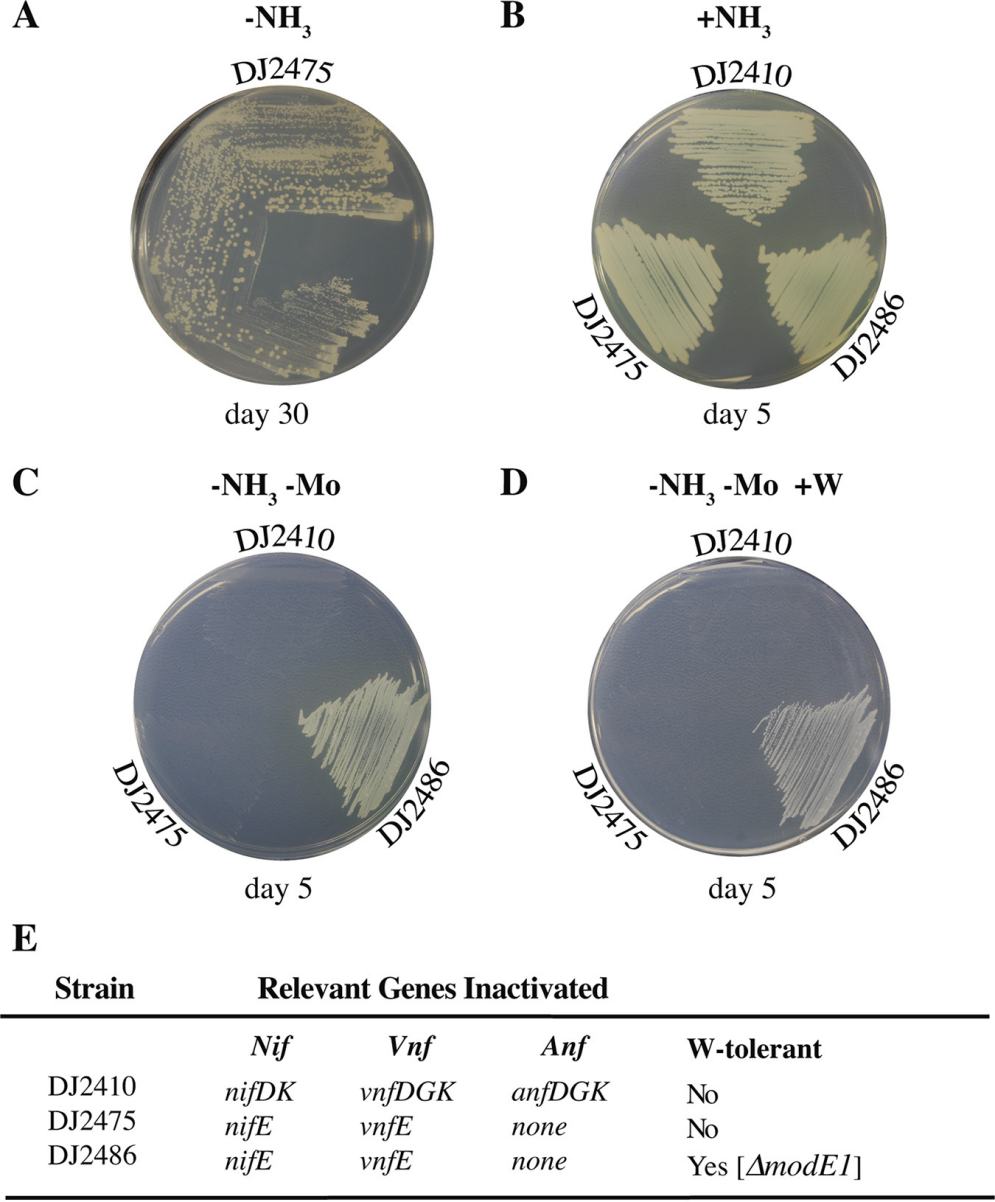
Inactivation of the *modE1* gene alleviates the delayed diazotrophic growth phenotype observed for DJ2475. (A) DJ2475 was incubated on Burks medium agar plates supplemented with Mo for 30 days to select for pseudorevertants, which can be recognized as large colonies. One pseudorevertant was selected and designated DJ2486. Sequence analysis of genomic DNA from DJ2486 revealed it carries a 1-bp deletion in *modE1* (Table S1) but no mutations within *modE2*. (B) All strains have the capacity for growth in medium supplemented with NH_3_ as a fixed nitrogen source. (C) The *modE1* mutation in DJ2486 rescues the delayed Fe-only-dependent diazotrophic growth phenotype evident in the parental DJ2475 strain. (D) In addition to curing the delayed diazotrophic growth phenotype, the *modE1* mutation carried in DJ2486 also results in W-tolerance. (E) Relevant genotypes (refer to Table S1 for complete genotypic descriptions).

Given that many genes are deleted in the CA6 W-tolerant A. vinelandii strain, it was of interest to ask if there is a single gene whose individual inactivation might alleviate the phenotype associated with the proposed repression of Fe-only nitrogenase accumulation by trace levels of Mo observed in the present work. This possibility was first explored by selection of pseudorevertants of strain DJ2475, inactivated for NifEN and VnfEN but having an intact Mo acquisition capacity, that exhibit rapid Fe-only nitrogenase-dependent diazotrophic growth (Fig. 10A). One pseudorevertant, designated DJ2486, was selected for further analysis, and the DNA sequences of *modE1* and *modE2* were determined. Both *modE1* and *modE2* were analyzed because it has been reported that both ModE1 and ModE2 are involved in the Mo-dependent repression of the Fe-only system and that inactivation of ModE1 disables high-affinity Mo acquisition (56, 59). This analysis revealed that the *modE2* gene remained intact in DJ2486 but that there is a 1-bp deletion located at nucleotide 306 in the *modE1* coding sequence. Thus, either *modE1*, or a gene located downstream of *modE1* whose expression is affected by the frameshift mutation, is responsible for alleviating delayed Fe-only nitrogenase-dependent diazotrophic growth recognized for DJ2475 (Fig. 10C). This issue was clarified by showing that the delayed Fe-only nitrogenase-dependent diazotrophic growth phenotype exhibited by another strain (DJ2479), also inactivated for both NifEN and VnfEN, could be rescued by placing an in-frame deletion within the *modE1* gene (DJ2491). Both strains inactivated for *modE1* (DJ2486 and DJ2491) could also grow diazotrophically in the presence of 1 mM W whereas strains DJ2475 and DJ2479 and the parental wild-type strain (DJ) exhibited no diazotrophic growth when cultured in the presence of 1 mM W (Fig. 10 and Fig. S2). In aggregate, these results are consistent with previous work that established that only trace levels of Mo are sufficient to repress accumulation of the Fe-only nitrogenase and that such repression can be partially alleviated by inactivation of *modE1* (58). Parenthetically, it was also observed that an otherwise wild-type strain, designated DJ2340 (Table S1), exhibits the W-tolerant phenotype when the *modE1* gene is inactivated, further indicating that inactivation of *modE1* is sufficient to relieve repression of the Fe-only nitrogenase expression under conditions used in the present work. Whether or not this effect is mediated directly by incapacitation of ModE1 repression of Fe-only nitrogenase or indirectly as a result of defective Mo acquisition remains to be explored.

## DISCUSSION

The key findings of the present work are that NifEN does not physiologically replace the function of VnfEN for the formation of FeV-cofactor and that neither NifEN nor VnfEN is involved in the formation of FeFe-cofactor. These results lead to the model shown in Fig. 2 wherein it is proposed that the specificity of heterometal incorporation (Mo or V) during the formation of FeMo-cofactor and FeV-cofactor is provided by the NifEN complex or VnfEN complex, respectively. Although NifH (Fe protein-1) and VnfH (Fe protein-2) are known to be involved in the corresponding cofactor maturation processes, they are unlikely to be directly responsible for metal composition specificity for the respective components because both *in vitro* and *in vivo* analyses have established that VnfH can replace the function of NifH in FeMo-cofactor maturation (32, 33). The present work also demonstrates that neither a NifEN nor a VnfEN assembly node is required for formation of FeFe-cofactor, indicating that, rather than being separately formed and inserted into an immature form of the FeFe protein, FeFe-cofactor assembly is completed within the FeFe protein. These findings are in agreement with the work of Yi-Ping Wang, Ray Dixon, and colleagues (36) and further highlight the Fe-only nitrogenase system as an attractive target for endowing a eukaryote with an ability to fix nitrogen. It is also interesting that the Fe-only system does not encode paralogs to either NifX/VnfX or NifY/NafY/VnfY. NifX and VnfX are proposed to traffic NifB-co to NifEN or VnfEN, respectively (60, 61) (Fig. 2). Similarly, NifY/NafY and VnfY are proposed to mediate transfer of FeMo-cofactor and FeV-cofactor between either NifEN and apo-MoFe protein or VnfEN and apo-VFe protein, respectively (62, 63). The lack of a NifY/NafY/VnfY counterpart within the Fe-only system makes sense because, based on the present work, FeFe-cofactor assembly is apparently completed within the immature form of the FeFe protein, so there is no need for a trafficking protein in this case. In the case of trafficking of NifB-co to the apo-form of the FeFe protein, one possibility is that this function could be supplied by the NifX/VnfX-like domain found in the C-terminal region of NifB (64). Bioinformatic analysis of 68 NifB primary structures from organisms known or predicted to express an Fe-only nitrogenase (47), excluding the *Archaea*, indicates that 93% of them include a NifX/VnfX domain. If the NifX/VnfX domain contained in NifB is indeed involved in NifB-co trafficking during maturation of the FeFe protein, this situation could further simplify the development of a robust nitrogen-fixing eukaryote on the basis of the Fe-only nitrogenase system. Finally, the conclusion that neither the NifEN nor VnfEN cofactor assembly scaffolds are required for maturation of the FeFe protein, as found in the present work, can be reconciled with a previous report concluding that either NifEN or VnfEN is required for FeFe-cofactor formation by genotypic differences of strains used in the two studies that appear to be related to Mo-dependent repression of the Fe-only system.

## MATERIALS AND METHODS

### Strains and plasmids.

Strains used in this study are listed in Table S1 in the supplemental material. Relevant genes that are affected by deletion and/or insertion mutations are indicated in the figures. The genetic pedigree of each strain can be traced using the designation of the parental strain used for mutagenesis in each case. Strains CA and CA11 have been previously described and were obtained from Paul Bishop (65, 66). The precise location of insertion and/or deletion mutations for each strain can be found in Table S2, which lists the plasmids used for each strain construction. Mutations were incorporated into the A. vinelandii genome by transformation as previously described (67). All plasmids used in the present work were ultimately derived from a ColE1-based vector and cannot replicate in A. vinelandii. Deletions and/or kanamycin/streptomycin resistance-encoding cartridge insertions were confirmed by PCR amplification of genomic DNA (68) using appropriate DNA primers and, in certain cases, sequence determination of amplified DNA. The isolation of strains DJ1254 and DJ1255, which carry the 42,096-bp deletion that bestows W tolerance and were ultimately used for many other strain constructions, was previously described in detail (39, 48). Strains that carry an affinity Strep tag having the sequence ASWSHPQFEK located at either the N terminus of VnfK or the C terminus of AnfD are indicated by an “S-Tag” superscript in Table S1. Strains DJ and UF were constructed in the Dean and Einsle laboratories, respectively.

### Growth.

A. vinelandii cells were grown at 30°C in Burks modified nitrogen-free medium plates (69). For nondiazotrophic conditions, ammonium acetate was added to the medium at a final concentration of 13 mM as the nitrogen source. Where indicated, molybdate (Na_2_MoO_4_) (J. T. Baker), metavanadate (NaVO_3_) (Sigma-Aldrich), or tungstate (Na_2_WO_4_) (Acros Organic) was added to the medium in a final concentration of 1 μM, 2 μM, or 1 mM, respectively. For large-scale cultures, A. vinelandii cells were grown in a 150-liter custom-built fermentor (W. B. Moore, Inc., Easton, PA) at 30°C in modified Burks medium containing 1 mM urea as nitrogen source. Cells were grown overnight and harvested at an optical density at 600 nm (OD_600_) of 1.6. Strains shown in Fig. 4 and 5 were cultured on agar plates for 5 days.

### Protein purification and analysis.

Strep-tagged proteins were purified following procedures previously described (30) using Strep-Tactin columns (IBA Lifesciences, Göttingen, Germany). Fe protein-3 was purified from DJ2303 following a previously published procedure (70) including some modifications; as a first step, the cell extract was passed over a Strep-Tactin column matrix to remove Strep-tagged FeFe protein. The flowthrough was then subjected to two NaCl Strep-gradients using DEAE column chromatography (Cytiva) followed by Q-Sepharose column chromatography (Cytiva). Fe protein-3 eluted from the Q-Sepharose column at 170 to 220 mM NaCl. Elutions containing the Fe protein-3 were concentrated using a Q-Sepharose column, and the brown protein was eluted and stored in liquid nitrogen. The purity of the proteins was determined by SDS-PAGE analysis. Protein concentrations were determined by the bicinchoninic acid (BCA) method (BCA protein assay kit; Sigma-Aldrich). Metal content (Fe, Mo, and V) was determined by inductively coupled plasma mass spectrometry (ICP-MS) (Metals Analysis Service, Virginia Tech).

### Substrate reduction assays.

Substrate reduction assays were conducted using sealed 9.4-ml serum vials as previously described (48). Vials contained an assay buffer consisting of a MgATP regeneration system (6.7 mM MgCl_2_, 30 mM phosphocreatine, 5 mM ATP, 0.2 mg/ml creatine phosphokinase, 1.2 mg/ml bovine serum albumin [BSA]) and 10 mM sodium dithionite in 100 mM morpholinepropanesulfonic acid (MOPS) buffer at pH 7.3. Solutions were made anaerobic, and headspace gases in the reaction vials were adjusted to the desired partial pressures of relevant gaseous substrates (1 atm N_2_ or 0.1 atm C_2_H_2_) per condition indicated. Any remaining space was filled with argon. After addition of 0.1 mg FeFe protein to each assay vial, the vials were ventilated to atmospheric pressure, and the reactions were initiated by the addition of AnfH (Fe protein-3) at a molar ratio of 30 Fe protein per 1 FeFe protein. Reaction mixtures were incubated at 30°C for 8 min and then quenched by the addition of 500 μl of 400 mM EDTA (pH 8.0). The products (NH_3_, H_2_, and C_2_H_4_) from the different substrate reduction assays were quantified according to published methods (71, 72) with minor modifications.

### EPR spectroscopy.

Continuous-wave X-band electron paramagnetic resonance (EPR) spectra were recorded using a Bruker ESP-300 spectrometer with an EMX PremiumX microwave bridge and an EMXplus standard resonator in perpendicular mode, equipped with an Oxford Instruments ESR900 continuous helium flow cryostat using a VC40 flow controller for helium gas. Spectra were recorded in 4-mm calibrated quartz EPR tubes (Wilmad LabGlass, Vineland, NJ) under the following conditions: temperature, 12 K; microwave frequency, 9.4 GHz; microwave power, 20 mW; modulation frequency, 100 KH_Z_; modulation amplitude, 8.14 G; time constant, 20.48 ms. The cavity background signal was recorded using an EPR tube filled with 100 mM MOPS buffer at pH 7.3 and was subtracted from the experimental spectra. Each spectrum represents the sum of 5 scans. Spectra presented were normalized to the same concentration of FeFe protein (10 mg/ml).

### Data availability.

Data that support the findings of this study are available within the article and its supplemental material.
