# Supplementary material for: Specificity of NifEN and VnfEN for the Assembly of Nitrogenase Active Site Cofactors in Azotobacter vinelandii
Source: mBio. 2021 Jul 20;12(4):e01568-21. doi: 10.1128/mBio.01568-21 (PMC8406325; doi:10.1128/mBio.01568-21)
Supplement: TABLE S1 [file mbio.01568-21-st001.docx]

**Table S1. List of *A. vinalendii* strains used in this work**.

The list includes the detailed genotypes for each strain used in this work. Information in the table permits tracing of the genealogy of each strain back to the original DJ and CA wild-type strains. Details of plasmids used for the construction of each strain can be found in Table S2. DJ2486 is a pseudorevertant originated from DJ2475. W^T^: tungsten-tolerant; H-TAG: His-tag; S-TAG: Strep-tag; km: kanamycin; sm: spectinomycin; gm: gentamycin.

**Strain Parent DNA Used Genotype**

DJ --- --- wild-type

CA --- --- wild-type

CA11 CA pDB11 Δ*nifHDKTY*

CA11.6 CA11 --- Δ*nifHDKTY*, Δ42kbp (W^T^)

DJ33 DJ pDB33 Δ*nifDK*

DJ200 DJ pDB253 Δ*nifDK*::km^R^

DJ995 DJ200 pDB827 *nifD*^H-TAG^

DJ1007 DJ995 pDB35 Δ*nifE*, *nifD*^H-TAG^

DJ1254 DJ33 CA11.6 Δ*nifDK*, Δ42kbp (W^T^)

DJ1255 DJ1254 pDB1087 Δ*nifDK*, Δ*vnfK*::km^R^, Δ42kbp (W^T^)

DJ2239 DJ1255 pDB2139 Δ*nifDK*, Δ*vnfDK*::sm^R^, Δ42kbp (W^T^)

DJ2240 DJ2239 pDB2134 Δ*nifDK*, Δ*vnfDGK*::sm^R^, Δ*anfDGK*::km^R^, Δ42kbp (W^T^)

DJ2241 DJ2240 pDB2158 Δ*nifDK*, Δ*vnfDGK*::sm^R^ *anfD* ^S-TAG^, Δ42kbp (W^T^)

DJ2245 DJ2241 pDB218 Δ*nifDK*, Δ*vnfDGK*::sm^R^ *anfD*^S-TAG^, Δ*nifB*::km^R^, Δ42kbp (W^T^)

DJ2253 DJ1255 pDB2187 Δ*nifDK*, *vnfK*^S-TAG^, Δ42kbp (W^T^)

DJ2254 DJ2253 pDB2134 Δ*nifDK*, Δ*anfDGK*::km^R^, *vnfK*^S-TAG^ Δ42kbp (W^T^)

DJ2303 DJ2241 pDB259 Δ*nifDK*, Δ*vnfDGK*::sm^R^, Δ*nifE*::km^R^, *anfD*^S-TAG^, Δ42kbp (W^T^)

DJ2379 DJ33 pDB2139 Δ*nifDK*, Δ*vnfDGK*::sm^R^

DJ2381 DJ2241 pDB2295 Δ*nifDK*, Δ*vnfDGK*::sm^R^, Δ*vnfEN*, *anfD*^S-TAG^ Δ42kbp (W^T^)

DJ2387 DJ2381 pDB259 Δ*nifDK*, Δ*vnfDGK*::sm^R^, Δ*nifE*::km^R^, Δ*vnfEN*, *anfD*^S-TAG^ Δ42kbp (W^T^)

DJ2410 DJ2379 pDB2308 Δ*nifDK*, Δ*vnfDGK*::sm^R^, Δ*anfDGK*::km^R^

DJ2425 DJ2410 pDB2158 Δ*nifDK*, *ΔvnfDGK*::sm^R^, *anfD*^S-TAG^

DJ2455 DJ2253 pDB2347 Δ*nifDK*, Δ*vnfE*::sm^R^, *vnfK*^S-TAG^

DJ2456 DJ2254 pDB2347 Δ*nifDK*, Δ*anfDGK*::km^R^, Δ*vnfE*::sm^R^, *vnfK*^S-TAG^

DJ2475 DJ1007 pDB2347 Δ*nifE*, Δ*vnfE*::sm^R^, *nifD*^H-TAG^

DJ2479 DJ2425 pDB2200 & pDB259 Δ*nifDK*, Δ*vnfDGK*::sm^R^, Δ*vnfEN*::gm^R^, Δ*nifE*::km^R^, anfD^S-TAG^

DJ2486 DJ2475 see legend Δ*nifE*, Δ*vnfE*::sm^R^, nifD^H-TAG^, Δnt-306 *modE1* (W^T^)

DJ2491 DJ2479 pDB2265 Δ*nifDK*, Δ*vnfDGK*::sm^R^, Δ*nifE*::km^R^, Δ*vnfEN*::gm^R^, anfD^S-TAG^ Δ*modE1* (W^T^)

UF63 DJ2241 pJG69 Δ*nifDK*, Δ*vnfDGK*::sm^R^, Δ*nifENX*::km^R^*, anfD* ^S-TAG^, Δ42kbp (W^T^)

UF64 DJ2241 pJG51 Δ*nifDK*, Δ*vnfDGK*::sm^R^, Δ*vnfENX*::km^R^, *anfD* ^S-TAG^, Δ42kbp (W^T^)

UF67 DJ pJG51 & pJG26 Δ*nifDK*, Δ*vnfDGK*::sm^R^, Δ*nifENX*::km^R^, Δ*vnfENX*::sm^R^
