# Supplementary material for: Specificity of NifEN and VnfEN for the Assembly of Nitrogenase Active Site Cofactors in Azotobacter vinelandii
Source: mBio. 2021 Jul 20;12(4):e01568-21. doi: 10.1128/mBio.01568-21 (PMC8406325; doi:10.1128/mBio.01568-21)
Supplement: TABLE S2 [file mbio.01568-21-st002.docx]

**Table S2. List of plasmids used for *A. vinelandii* strain construction.**

Location of residues removed and/or placement of insertions are indicated.

**Plasmid Deletion/Insertion Residues Removed/**

**Insertion Location**

pDB11 Δ*nifHDKTY* NifH^70^ – NifY^14^

pDB33 Δ*nifDK* NifD^103^ – NifK^308^

pDB35 Δ*nifE* NifE^133-389^

pDB218 Δ*nifB*::km^R^ NifB^60 - 307^

pDB253 Δ*nifDK*::km^R^ NifD^476^-NifK^294^

pDB259 Δ*nifE*::km^R^ NifE^15 - 261^

pDB827 *nifD*::*H-TAG* NifD^481-H-TAG^

pDB1087 *vnfK*::km^R^ VnfK^2^

pDB1124 *anfD*::km^R^ AnfD^4^

pDB2134 Δ*anfDGK* AnfD^204^ - AnfK^148^

pDB2139 Δ*vnfDGK*::sm^R^ VnfD^271^ - VnfK^202^

pDB2158 *anfD*::*S-TAG* AnfD^518^

pDB2187 *vnfK*::*S-TAG* VnfK^2-S-TAG^

pDB2200 *vnfE*::gm^R^ VnfE^91^

pDB2265 Δ*modE1* ModE1^151 - 215^

pDB2295 Δ*vnfEN* VnfE^67^ - VnfN^414^

pDB2308 Δ*anfDGK*::km^R^ AnfD^204^ - AnfK^148^

pDB2347 Δ*vnfE*::smR VnfE^30 – 462^

pJG26 Δ*vnfENX*::smR VnfE^1^ - VnfX^182^

pJG51 ΔvnfENX::km^R^ VnfE^1^ - vnfX^182^

pJG69 Δ*nifENX*::km^R^ NifE^1^ - NifX^159^
