# Supplementary figures and images for: Specificity of NifEN and VnfEN for the Assembly of Nitrogenase Active Site Cofactors in Azotobacter vinelandii
Source: mBio. 2021 Jul 20;12(4):e01568-21. doi: 10.1128/mBio.01568-21 (PMC8406325; doi:10.1128/mBio.01568-21)

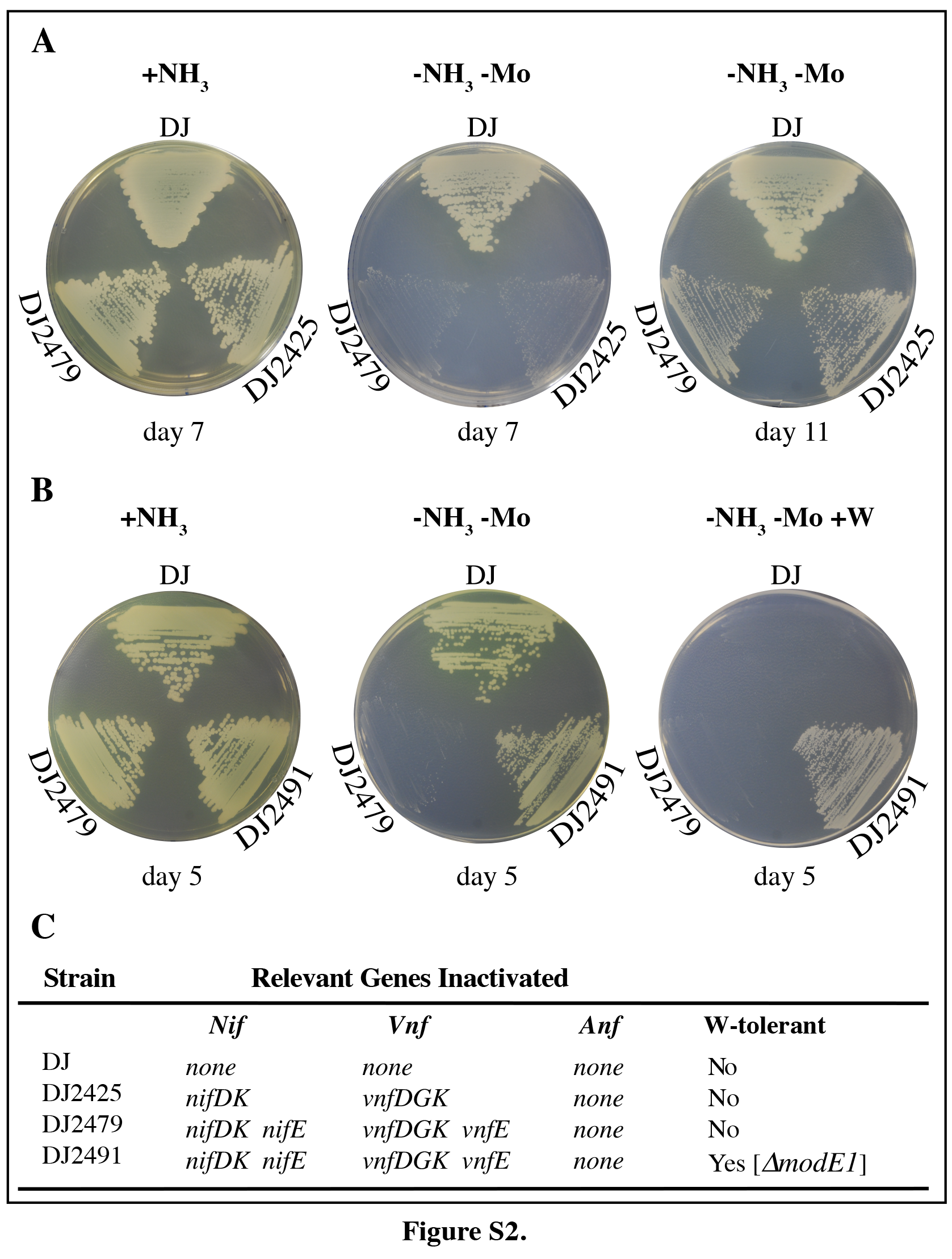

Supplement: FIG S2 [file mbio.01568-21-sf002.tif]
